# Alternative splicing of *ALCAM* enables tunable regulation of cell-cell adhesion through differential proteolysis

**DOI:** 10.1038/s41598-018-21467-x

**Published:** 2018-02-16

**Authors:** Katie E. Hebron, Elizabeth Y. Li, Shanna A. Arnold Egloff, Ariana K. von Lersner, Chase Taylor, Joep Houkes, David K. Flaherty, Adel Eskaros, Thomas P. Stricker, Andries Zijlstra

**Affiliations:** 1 Vanderbilt University, Program in Cancer Biology, Nashville, USA; 2 Department of Biology, Massachusetts Institute of Technology, Cambridge, USA; 3 Department of Pathology, Microbiology and Immunology, Vanderbilt University Medical Center, Nashville, USA; 4 Department of Veterans Affairs, Tennessee Valley Healthcare System, Nashville, USA; 5 Department of Microbiology, Wageningen University and Research, Wageningen, Netherlands; 6 Vanderbilt University Medical Center, Vanderbilt Vaccine Center, Nashville, USA

## Abstract

While many adhesion receptors are known to influence tumor progression, the mechanisms by which they dynamically regulate cell-cell adhesion remain elusive. We previously identified Activated Leukocyte Cell Adhesion Molecule (ALCAM) as a clinically relevant driver of metastasis and hypothesized that a tunable mechanism of ectodomain shedding regulates its contribution to dissemination. To test this hypothesis, we examined an under-explored *ALCAM* splice variant (ALCAM-Iso2) and demonstrated that loss of the membrane-proximal region of ALCAM (exon 13) increased metastasis four-fold. Mechanistic studies identified a novel MMP14-dependent membrane distal cleavage site in ALCAM-Iso2, which mediated a ten-fold increase in shedding, thereby decreasing cellular cohesion. Importantly, the loss of cohesion is not limited to the cell capable of shedding because the released extracellular domain diminished cohesion of non-shedding cells through disruption of ALCAM-ALCAM interactions. ALCAM-Iso2-dominated expression in bladder cancer tissue, compared to normal bladder, further emphasizes that *ALCAM* alternative splicing may contribute to clinical disease progression. The requirement for both the loss of exon 13 and the gain of metalloprotease activity suggests that ALCAM shedding and concomitant regulation of tumor cell adhesion is a locally tunable process.

## Introduction

Dynamic control of cell-cell adhesion is central to many normal biological processes, including neuronal guidance, cell differentiation, tissue morphogenesis, and immune cell activation and function (reviewed in ref.^1^). Dysregulation of cell-cell adhesion can lead to pathologies of the muscle, skin, and kidney, as well as the nervous and immune systems (reviewed in ref.^2^). In other diseases, such as cancer, the dysregulation of adhesion is a central mediator of malignant progression that can support not only invasion and dissemination but also cell survival and proliferation.

All major classes of adhesion molecules have been shown to contribute to cancer progression. For example, loss of epithelial cadherin (E-cadherin) expression is a canonical indication of changing cell-cell adhesions that facilitate motility during oncogenic transformation^3^, while changes in integrin expression correlate with tumor progression, metastasis, and chemoresistance^4–7^. Additionally, following the loss of E-cadherin, the immunoglobulin superfamily of cell adhesion molecules (Ig-CAMs) is upregulated in tumor cells where it modulates cellular proliferation and survival, while promoting disease progression through modulation of matrix metalloprotease (MMP) expression, collective cell migration, and tumor cell-endothelial cell interactions^8–11^. While changes in the expression of these adhesion receptors have been associated with tumor progression, the mechanisms underlying dynamic regulation of their activity remain poorly understood.

The Ig-CAM, Activated Leukocyte Cell Adhesion Molecule (ALCAM), has been shown to modulate cell-cell adhesion in two distinct fashions, through homotypic ALCAM-ALCAM interactions and through heterotypic ALCAM-CD6 interactions. In normal physiology, homotypic ALCAM interactions modulate cell-cell interactions of epithelial and endothelial cells and mediate neuronal guidance, while ALCAM-CD6 interactions are essential for antigen presentation in immune cell adhesion^12–15^. ALCAM is also essential for monocyte transendothelial cell migration specifically in the brain, but it is currently unknown whether this function can be attributed to homotypic or heterotypic ALCAM interactions^16^.

In cancer, ALCAM has emerged as a significant factor in disease progression; however, the relationship between the expression of ALCAM and its correlation with aggressive disease has been debated. Changes in ALCAM subcellular localization from the cell surface to the cytoplasm in breast cancer correlate with poor prognosis^17^. However, loss of ALCAM by immunohistochemistry correlates with advanced stage in prostate and bladder cancer, but the loss of ALCAM protein in the tumor tissue is inconsistent with the persistent and sometimes elevated expression of ALCAM mRNA^18,19^. Finally, ALCAM expression positively correlates with increased tumorigenicity and invasiveness in melanoma, pancreatic cancer, and liver cancer^20–22^. Additional mechanistic studies support the role of ALCAM in promoting tumor progression. It has been shown to promote survival in breast cancer cells through the anti-apoptotic protein B-cell lymphoma 2 (Bcl-2), modulate invasion of melanoma through expression of MMP2 and MMP14, and promote metastasis through collective cell invasion^9,23,24^. Despite this large body of evidence indicating that ALCAM is important to cancer progression, the mechanism by which ALCAM contributes to tumor progression remains unclear.

In the absence of evidence for an activation mechanism, such as phosphorylation, the regulation of ALCAM-mediated adhesion is thought to be achieved by controlling ALCAM binding availability. Regulation of ALCAM-ALCAM interactions can occur through changes in expression and membrane localization, proteolytic release of the ALCAM ectodomain, or antagonistic competition by this shed ectodomain and/or a soluble ALCAM isoform^9,10,17,25^. ALCAM, like many other Ig-CAMs, can be cleaved from the cell surface by the protease ADAM17, a process referred to as ectodomain shedding^9,26^. However, the regulation of this process is not well characterized. Work from our laboratory and that of others has demonstrated that the level of shed ALCAM in biofluids is elevated in a variety of cancer patients, suggesting that ALCAM shedding increases during oncogenesis and malignant progression^19,26,27^. As previously noted, changes in total mRNA expression rarely correlate with the changes in protein expression in tumor tissues, therefore suggesting that ALCAM shedding is the likely cause for these discrepancies reported in the literature. Thus, we aimed to elucidate the intrinsic regulatory mechanisms controlling ALCAM shedding in cancer progression.

A review of the gene structure of *ALCAM* revealed the existence of a splice variant missing the membrane proximal stalk region, which is targeted by ADAM17 cleavage. This led us to hypothesize that dynamic changes in ectodomain shedding might be achieved through changes in protease susceptibility between *ALCAM* splice isoforms. *ALCAM* has nine recognized splice variants, four of which have open reading frames (Ensembl^28^). However, only two isoforms have been confirmed on both mRNA and protein levels (Sup. Table ST1). Full length ALCAM (ALCAM-Iso1) is composed of all 15 coding exons. ALCAM-Iso2 lacks exon 13, which corresponds to 13 amino acids in the stalk region of the protein (Sup. Fig. S1a, Ensembl^28^). Despite the fact that both isoforms are expressed in normal human tissues (Sup. Fig. S1b), biochemical and functional differences between the two isoforms have never been explored.

In this manuscript, we examined the functional and biochemical differences between ALCAM-Iso1 and ALCAM-Iso2 in the context of tumor progression. We investigated the effect of ALCAM-Iso1 and ALCAM-Iso2 expression on metastasis, tumor cell aggregation, and ALCAM homotypic interactions. We defined differences in proteolytic susceptibility between ALCAM-Iso1 and ALCAM-Iso2, as well as the impact of isoform-dependent proteolytic susceptibility on cell-cell adhesion. Finally, we conclude that differential expression of ALCAM-Iso1 and ALCAM-Iso2 correlates with malignancy in bladder tissue.

## Results

### Alternative splicing of *ALCAM* (ALCAM-Iso2) promotes metastatic dissemination

**Figure 1:**
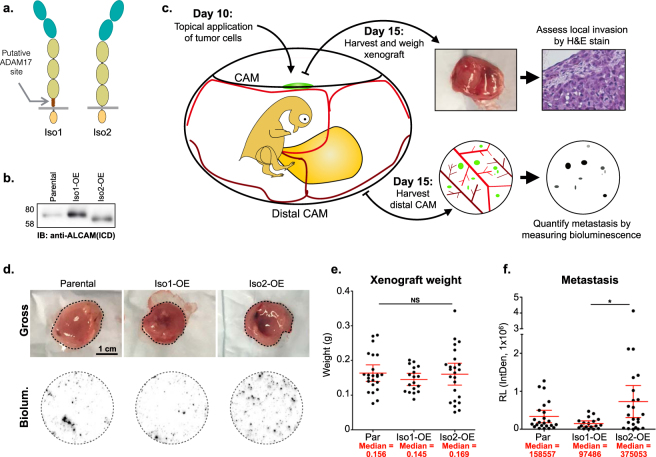
Alternative splicing of *ALCAM* (ALCAM-Iso2) promotes metastatic dissemination. (**a**) Schematic describing ALCAM splice isoforms 1 and 2. (**b**) Immunoblot of parental HT1080 cells (Par) and HT1080 cells stably overexpressing ALCAM-Iso1 (Iso1-OE) or ALCAM-Iso2 (Iso2-OE); ICD: intracellular domain. (**c**) Schematic describing avian embryo spontaneous metastasis assay. (**d**) Representative images of gross (tumor is outlined in dashed line) HT1080 xenografts and metastasis to the distal chorioallantoic membrane (CAM), indicated by bioluminescence (Biolum.). (**e**) Wet weight of HT1080 xenografts removed from CAM of chicken embryos. Three independent experiments were combined for the analysis (N = 23, 18, 25, respectively). (**f**) Quantification of metastasis. Metastasis was measured by luciferase activity in a 1.5 cm core of distal CAM. Two cores per embryo from at least 10 embryos were measured. Relative bioluminescence (RL) represents mean value of two CAM sections per chick and was calculated using the integrated density tool (IntDen, 1 × 10^6^) in ImageJ. (**e**,**f**) P-values were calculated using Kruskal-Wallis test with Dunn’s post-test, ns: not significant, *P < 0.05. Graphs display mean with 95% confidence interval. Medians are reported.

ADAM17 is known to cleave its substrates in the “stalk region”, which is the extracellular region between the transmembrane motif and the next extracellular globular region of the protein^29–31^. In ALCAM-Iso1, the stalk region corresponds to amino acids 502–527^32^ encoded by exons 12–14. Alternative splicing of exon 13 in ALCAM-Iso2 removes the majority of the stalk region (13 amino acids), which could impact the proteolytic processing of ALCAM-Iso2 (Fig. 1a, Sup. Fig. S1a) and therefore alter its adhesive function^26,33^. Since ALCAM ectodomain shedding is elevated in patients with advanced disease^19,26,27^ and both isoforms are present in many tissues (Sup. Fig. S1b), we hypothesized that alternative splicing could impact metastatic dissemination. To test this hypothesis, we generated a panel of HT1080 cell lines in which expression of the ALCAM isoforms was experimentally controlled (Sup. Fig. S2). Overexpression (OE) of ALCAM-Iso1 or ALCAM-Iso2 (Iso1- and Iso2-OE, Fig. 1b, Sup. Fig. S2a) generated a modest increase in surface ALCAM compared to levels observed in the parental HT1080 cells (Sup. Fig. S2b). Using the avian embryo spontaneous metastasis assay (Fig. 1c)^34–36^ no differences in xenograft size were observed (Fig. 1d,e). However, overexpression of ALCAM-Iso2 showed increased incidence of intravascular invasion, as scored by a pathologist (Sup. Fig. S3), with a concomitant, four-fold increase in spontaneous metastasis compared to overexpression of ALCAM-Iso1, as quantified by bioluminescent detection of tumor cells (Fig. 1d,f).

**Figure 2:**
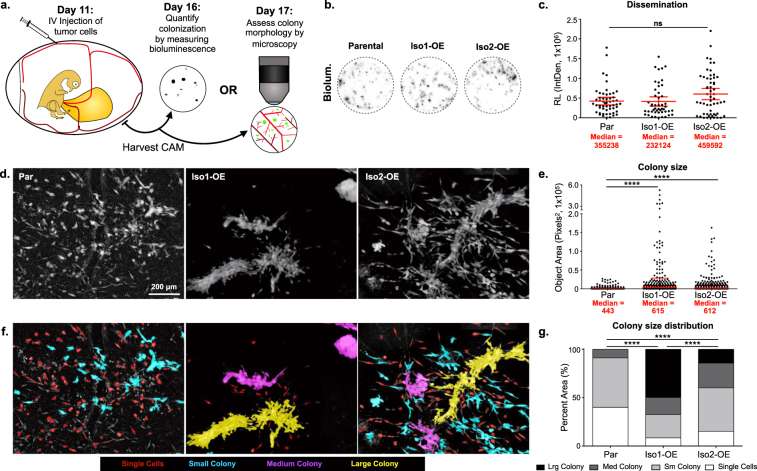
Full length ALCAM (ALCAM-Iso1) mediates tumor cell cohesion. (**a**) Schematic describing avian embryo experimental metastasis assay. (**b**) Representative images of CAM colonization, measured by bioluminescence, following intravenous (IV) injection of indicated HT1080 cells. (**c**) CAM colonization was quantified using luciferase activity in a 1.5 cm core of CAM. Two cores per embryo from at least 10 embryos were measured. Relative bioluminescence (RL) represents mean value of two CAM sections per chick and was calculated using the integrated density tool (IntDen, 1 × 10^6^) in ImageJ. P-values were calculated using Kruskal-Wallis test with Dunn’s post-test, ns: not significant. (**d**) Representative images of metastatic colonies imaged 6 days post intravenous injection into CAM of chicken embryo. (**e**) Colony size was quantified using custom KNIME workflow. P-values were calculated using Kruskal-Wallis test with Dunn’s post-test, ****P < 0.0001. Graphs display mean with 95% confidence interval. Medians are reported. (**f**) Colonies were binned into single cell, small, medium, and large colonies using KNIME. Colonies are pseudo colored by size bin: Single Cells (Red), Small colonies (Cyan), Medium Colonies (Magenta), and Large colonies (Yellow). (**g**) Distribution of colonies across size bins was represented as percent of total area of all colonies. P-values were calculated using Chi-square test for trend, ****P < 0.0001. Graphs display mean with 95% confidence interval. Medians are reported.

To distinguish defects in distant site colonization from defects in primary tumor dissemination, ALCAM-mediated changes in colonization were evaluated in the avian embryo experimental metastasis model, where colony formation within the chorioallantoic membrane (CAM) is evaluated following intravenous injection (Fig. 2a). Quantification of colonization by bioluminescent activity revealed no significant difference in the number of colonies formed by parental, Iso1-OE, or Iso2-OE cells (Fig. 2b,c). These observations suggest that alternative splicing of *ALCAM* alters metastatic success by contributing to dissemination from the primary tumor.

### Full length ALCAM (ALCAM-Iso1) mediates tumor cell cohesion

While growth of metastatic colonies was not diminished, close examination of colony morphology revealed dense cell-cell cohesion among cells overexpressing ALCAM-Iso1 (Iso1-OE, Fig. 2d–g). In this model, differences in colony morphology correlate well with cell adhesion and metastasis^35,37,38^. High magnification *ex vivo* imaging of metastatic colonies formed in the CAM after intravenous injection revealed dense, multicellular colonies formed by HT1080 cells overexpressing ALCAM-Iso1 (Iso1-OE) while the colonies of the parental line were comprised of dispersed, single cells (Fig. 2d). Conversely, HT1080 cells overexpressing ALCAM-Iso2 (Iso2-OE) formed colonies composed of both diffuse single cells as well as densely-packed, cohesive cell colonies (Fig. 2d). Unbiased quantification of colony size confirmed a five-fold increase in cohesive colonies formed by Iso1-OE cells compared to parental cells (Fig. 2e). Each colony was subsequently classified as “Single Cell”, “Small Colony”, “Medium Colony”, and “Large Colony” based on its pixel area. This classification was pseudo-colored in the original image for visualization (Fig. 2f) and significant differences were assessed by Chi-squared test for trend (Fig. 2g). This analysis revealed that Iso1-OE cells primarily formed large colonies (50%) while the parental cells formed mostly single cells and small colonies (91%). In contrast to Iso1-OE, Iso2-OE cells formed few large colonies (14%) and persisted primarily as small colonies and dispersed single cells (61%), similar to the parental cells (Fig. 2g). These data suggest that ALCAM-Iso1 promotes cell-cell adhesion while ALCAM-Iso2 enables single cell dispersion.

### Alternative splicing of *ALCAM* leads to enhanced proteolytic susceptibility

**Figure 3:**
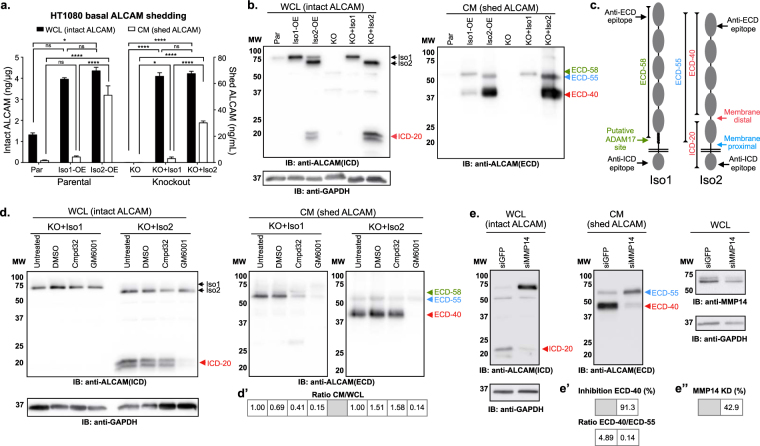
Alternative splicing of *ALCAM* introduces sensitivity to MMP14-dependent shedding. (**a**) ALCAM ELISA for quantification of basal extracellular domain shedding in parental HT1080 cells (Par) overexpressing ALCAM-Iso1 (Iso1-OE) or ALCAM-Iso2 (Iso2-OE), as well as in ALCAM-KO HT1080 cells (KO) rescued with either ALCAM-Iso1 (KO + Iso1) or ALCAM-Iso2 (KO + Iso2). ALCAM expression was measured in whole cell lysate (WCL) and shedding was measured in conditioned medium (CM). P-values were calculated using Kruskal-Wallis test with Dunn’s post-test; ns: not significant, *P < 0.05, ****P < 0.0001. (**b**) Immunoblot analysis of ALCAM expression and basal extracellular domain shedding in indicated cells. ICD: intracellular domain, ECD: extracellular domain. ALCAM fragments generated by shedding are marked as follows: 20 kDa intracellular domain fragment (ICD-20, ), 58 kDa extracellular domain fragment (ECD-58, ), 55 kDa extracellular domain fragment (ECD-55, ), and 40 kDa extracellular domain fragment (ECD-40, ). (**c**) Schematic of antibody epitopes and cleavage sites in ALCAM-Iso1 and ALCAM-Iso2. (**d**) Immunoblot analysis of extracellular domain shedding of ALCAM-KO HT1080 cells expressing ALCAM-Iso1 (KO + Iso1) or ALCAM-Iso2 (KO + Iso2) treated with an ADAM17-specific inhibitor, Compound 32 (Cmpd32, 10 μM), or a global metalloprotease inhibitor, GM6001 (10 μM). (d’) Changes in shedding were quantified as a ratio between intact ALCAM (WCL), and shed ALCAM (CM), with the untreated control normalized to 1. (**e**) Immunoblot analysis of extracellular domain shedding of ALCAM-KO HT1080 cells expressing ALCAM-Iso2 (KO + Iso2) transfected with anti-MMP14 siRNA. Results are representative of two independent experiments. (e’) Changes in shedding were quantified as percent decrease in ECD-40 band density compared to control (siGRP), or ratio of ECD-40 density to ECD-55 density. (e”) Knockdown (KD) of MMP14 was quantified as percent decrease in band density compared to control (siGRP). (**b**,**d**,**e**) 5 min exposures are shown. Individual blots are outlined in black. WCL blots were redeveloped with HRP-tagged anti-GAPDH for loading control. Blots have been cropped to show relevant bands, full-length blots are available in Supplementary Figure S7. (d’,e’,c’) Samples were derived from the same experiment. Gels and blots were processed in parallel.

Because ALCAM-Iso2 is generated through the loss of 13 amino acids in the stalk region of the protein, we hypothesized that the functional difference between these two isoforms may be driven by changes in ADAM17-mediated shedding of the ectodomain. To assess this, shedding of ALCAM-Iso1 and ALCAM-Iso2 was quantified by ELISA, with detection of intact ALCAM in the whole cell lysate (WCL) and shed ALCAM in the conditioned medium (CM). Unexpectedly, ectodomain shedding from Iso1-OE cells was ten-fold lower than that from Iso2-OE cells, despite nearly equal expression of ALCAM-Iso1 and ALCAM-Iso2 (Fig. 3a). To verify that the proteolytic susceptibility was a function intrinsic to the isoforms, rather than dominant-negative or dominant-active interactions between the isoforms, CRISPR/Cas9-mediated ALCAM-KO cells (KO) were generated along with re-expression of either ALCAM-Iso1 or ALCAM-Iso2 (KO + Iso1 and KO + Iso2, respectively, Sup. Fig. S2). The difference in ALCAM shedding was recapitulated in KO + Iso1 and KO + Iso2 cells, demonstrating that the increased susceptibility to proteolysis is intrinsic to the alternative splicing of *ALCAM* (Fig. 3a).

Analysis by immunoblotting with antibodies specific to the intracellular domain (ICD) and extracellular domain (ECD) of ALCAM was subsequently used to assess intact ALCAM in the WCL and shed ALCAM in the CM, respectively (Fig. 3b). This not only confirmed that the loss of exon 13 in ALCAM-Iso2 led to an increase in the proteolytic sensitivity, but also showed a change in the proteolytic profile, as evidenced by the production of two distinct soluble fragments (Fig. 3b, right panel). CM from KO + Iso1 cells contains a single ECD of relative molecular weight (rMW) 58 kDa (ECD-58), which corresponds to the expected ALCAM-ECD shed by ADAM17 (Fig. 3b, right panel). However, CM from Iso2-OE and KO + Iso2 cells contain the predicted ECD of rMW 55 kDa (ECD-55), as well as a second, more abundant ECD fragment of rMW 40 kDa (ECD-40) (Fig. 3b, right panel). While the residual ICD generated by ECD shedding from ALCAM-Iso1 is undetectable, the appearance of a new intracellular fragment of rMW 20 kDa (ICD-20) in the WCL from ALCAM-Iso2 indicates that the alternative splicing has exposed a novel proteolytic site (Fig. 3b, left panel). Based on relative molecular weights, this cleavage is predicted to occur at a site distal to the membrane in the fourth IgG-like domain (Fig. 3c).

### Alternative splicing of *ALCAM* introduces sensitivity to MMP-14 dependent shedding

Considering that ADAM17 has been identified as the sheddase of ALCAM-Iso1, we performed a directed protease inhibitor screen to determine if this protease was also responsible for the shedding of ECD-40 from ALCAM-Iso2. Following treatment with the indicated protease inhibitors, immunoblotting for ALCAM was performed in WCL and CM with antibodies directed to the ICD and ECD, as described above. Changes in shedding were quantified as a ratio between intact ALCAM, detected in the WCL, and shed ALCAM, detected in the CM, with the untreated control normalized to one (1). Treatment of KO + Iso1 with the ADAM17-specific inhibitor (Cmpd32) or a broad-spectrum metalloprotease inhibitor (GM6001) inhibited ALCAM-Iso1 ectodomain shedding (ECD-58) by 60% and 90%, respectively (Fig. 3d,d’, right panel). This level of inhibition is consistent with previous publications^26,39^. Conversely, the novel proteolytic cleavage of ALCAM-Iso2 that generated ECD-40 was inhibited by GM6001 but not by Cmpd32 (Fig. 3d,d’, right panel). The loss of ICD-20 from the WCL upon treatment with GM6001, but not with Cmpd32 (Fig. 3d, left panel), suggests that ICD-20 and ECD-40 are indeed the product of the same novel cleavage event. The activity of other classes of proteases against ALCAM-Iso2 was analyzed with a panel of broad spectrum protease inhibitors targeting metallo-, serine, cysteine, aspartyl, and trypsin-like proteases (Sup. Table ST2). ALCAM-Iso2 shedding was only inhibited by the broad-spectrum metalloprotease inhibitor GM6001 (Sup. Fig. S4).

Because the novel cleavage site was more distal to the typical juxtamembrane ADAM sites, we focused on matrix metalloproteases (MMP) as the most likely candidate metalloprotease family. MMP14, also known as membrane-type MMP-1 (MT1-MMP), was the top candidate because ALCAM expression is associated with the upregulation of MMP14 expression and subsequent MMP2 activation^24^. Specific siRNA-mediated knockdown of MMP14 in KO + Iso2 cells significantly decreased the generation of ECD-40 in the conditioned medium (91%), indicating that MMP14 is a novel participant in ALCAM-Iso2 ectodomain shedding (Fig. 3e,e’, right panel). Taken together, these data confirm that ALCAM-Iso1 is shed by ADAM17 through membrane-proximal cleavage in the stalk region, while ALCAM-Iso2 is predominantly shed in an MMP14-dependent manner at a novel, membrane-distal proteolytic site.

### Alternative splicing of *ALCAM* diminishes ALCAM-ALCAM adhesion

**Figure 4:**
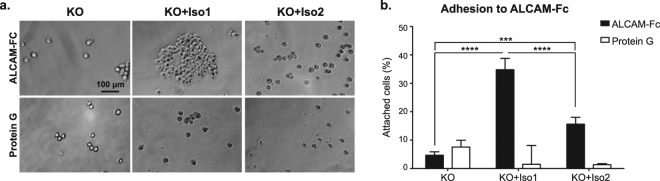
Alternative splicing of *ALCAM* diminishes ALCAM-ALCAM adhesion. (**a**) Representative images of ALCAM-KO HT1080 cells (KO) expressing ALCAM-Iso1 (KO + Iso1) or ALCAM-Iso2 (KO + Iso2) adhered to immobilized ALCAM-Fc. (**b**) Cells attached to ALCAM-Fc were stained with crystal violet dye. Percent of cells attached at 4 hours was quantified compared to cells attached to collagen type I coated wells. P-values were calculated using Kruskal-Wallis test with Dunn’s post-test, ***P < 0.001, ****P < 0.0001.

To further assess the functional consequences of this alternatively-spliced *ALCAM* isoform, we examined ALCAM-dependent adhesion *in vitro*. HT1080 ALCAM-knockout (KO) cells expressing ALCAM-Iso1 (KO + Iso1) or ALCAM-Iso2 (KO + Iso2) were seeded onto ALCAM-Fc directionally adhered to protein G-coated surfaces (Fig. 4a). KO + Iso1 cells adhered to ALCAM-Fc two-fold more than KO + Iso2 cells (Fig. 4b), suggesting that the differences in cell-cell adhesion in Iso1-OE cells and Iso2-OE cells are caused by differences in homotypic ALCAM interactions.

### Isoform-specific differences in cell-cell adhesion are controlled by ectodomain shedding

**Figure 5:**
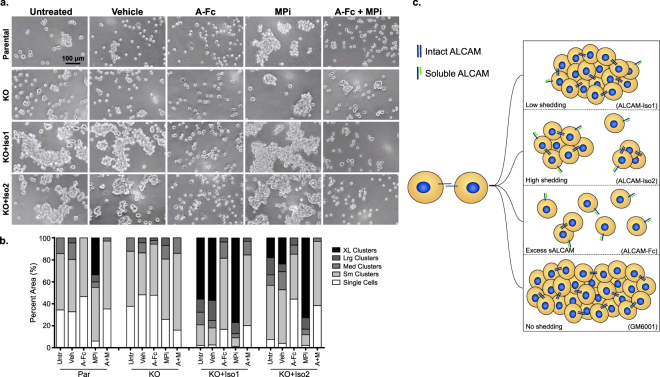
Isoform-specific differences in cell-cell adhesion are controlled by ectodomain shedding. (**a**) Representative images of *in vitro* cell aggregation analysis of parental HT1080 (Par) and ALCAM-KO HT1080 cells (KO) expressing ALCAM-Iso1 (KO + Iso1) or ALCAM-Iso2 (KO + Iso2). Veh: vehicle (0.4% DMSO), A-Fc: soluble ALCAM-Fc (10 μg/mL), MPi: metalloprotease inhibitor (GM6001, 10 μM). (**b**) Quantification of *in vitro* cell aggregation assay. Clusters were binned into single cell, small (Sm), medium (Med), large (Lrg) or extra-large (XL) clusters based on pixel area. Distribution of clusters across size bins was represented as percent of total area of all clusters. P-values were calculated using Chi-square test for trend and are listed in Supplementary Table ST3. Results are representative of three independent experiments. (**c**) Diagram representing aggregation phenotypes observed in indicated conditions. sALCAM: soluble ALCAM.

The enhanced colony cohesion *in vivo* (Fig. 2) and enhanced ALCAM-ALCAM adhesion of ALCAM-Iso1 *in vitro* (Fig. 4) suggests that the differential shedding between ALCAM-Iso1 and ALCAM-Iso2 (Fig. 3) controls the cell-cell adhesion function of ALCAM. To test this hypothesis, we evaluated *ALCAM* splicing and proteolysis-dependent changes in cell-cell adhesion using a cell aggregation assay (Fig. 5). Representative phase contrast images taken at 100X magnification are shown (Fig. 5a). Unbiased automated image analysis was used to quantify the cell cluster size. Similar to our *in vivo* colony size analysis, we analyzed cell cluster size distribution within each group (Fig. 5b) and used Chi-square analysis to define the significance of the changes across groups (Sup. Table ST3).

Untreated parental HT1080 cells and ALCAM-KO cells maintained for 12 h in suspension persisted primarily as a mixture of single cells (34% and 37%, respectively) and small clusters (51% and 50%, respectively) with a few medium size clusters (14% and 12%, respectively) but no large or extra-large clusters (Fig. 5a,b). Expression of ALCAM-Iso1 greatly enhanced cohesion and caused untreated KO + Iso1 cells to form mostly large and extra-large clusters (78%) with few small clusters and single cells (21%) (Fig. 5a,b). Conversely, the expression of ALCAM-Iso2 caused a significantly smaller portion of the untreated KO + Iso2 cells to form large and extra-large clusters (18%), with the majority of cells persisting as small clusters and single cells (57%, Fig. 5a,b, p < 0.0001, *Untreated*, Sup. Table ST3).

Disruption of ALCAM-ALCAM interactions by incubation with soluble recombinant ALCAM-Fc diminished aggregation by significantly inhibiting the formation of medium, large and extra-large clusters in all cell lines (Fig. 5a,b, *A-Fc*, P = 0.0022 -<0.0001, Sup. Table ST3), except in ALCAM-KO cells, where aggregation was already at a minimum (Fig. 5a,b, *A-Fc*). Preventing ALCAM shedding with a metalloprotease inhibitor (MPi; GM6001) promoted cell-cell adhesion leading to the formation of large and extra-large clusters in parental cells (from 0% to 35%), while ALCAM-KO cells had an increase in large clusters (from 0% to 7%), but no extra-large clusters with MPi treatment (Fig. 5a,b, *MPi*, P < 0.0001 and P = 0.0141, respectively, Sup. Table ST3). Conversely, aggregation in KO + Iso1 cells was enhanced modestly (1.4-fold for extra-large clusters) when shedding was inhibited by GM6001 (Fig. 5a,b, P = 0.0004, Sup. Table ST3). Unlike KO + Iso1 cells, aggregation in KO + Iso2 cells was strongly increased by treatment with GM6001 (four-fold for extra-large clusters, Fig. 5a,b, p < 0.0001, Sup. Table ST3). All of these findings were recapitulated in parental HT1080 cells overexpressing ALCAM-Iso1 or -Iso2 (Iso1-OE and Iso2-OE, respectively, Sup. Fig. S5, Sup. Table ST4). Finally, upon co-treatment with ALCAM-Fc, the clustering in response to MPi treatment was significantly diminished, resulting in dispersed single cells and small clusters for all cell lines, except ALCAM-KO cells, further supporting that this is an ALCAM-specific phenotype (Fig. 5a,b, *A-Fc* + *MPi*, P < 0.0001, Sup. Table ST3). Together, these data support our hypothesis that alternative splicing of *ALCAM* increases the sensitivity of ALCAM-Iso2 to proteolysis, leading to increased shedding and isoform-specific differences in cell-cell cohesion (Fig. 5c).

### Shed ALCAM-Iso2 can disrupt ALCAM-Iso1-mediated cell-cell adhesion in paracrine-like manner

**Figure 6:**
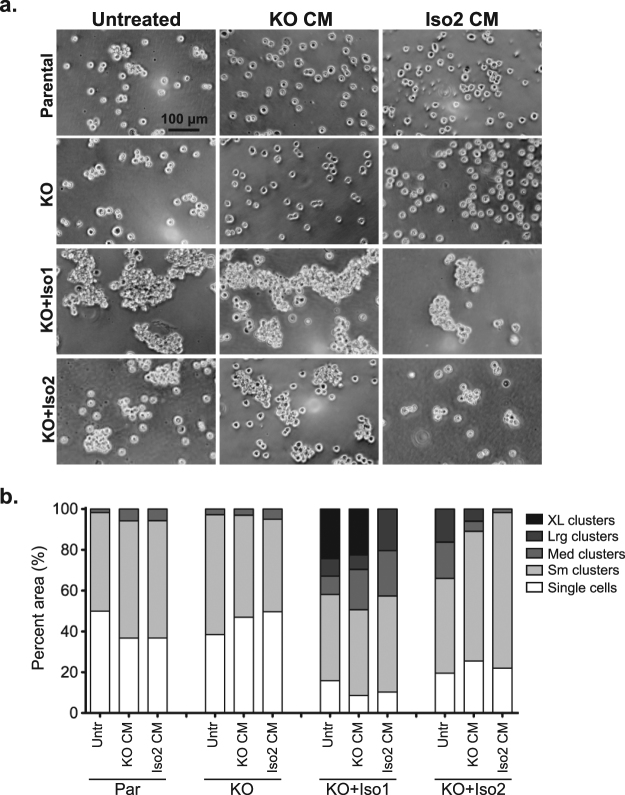
The shedding of alternatively spliced *ALCAM* (ALCAM-Iso2) can disrupt cell-cell adhesion mediated by full-length ALCAM (ALCAM-Iso1) in paracrine-like manner. (**a**) Representative images of *in vitro* cell aggregation analysis of parental HT1080 (Par) and ALCAM-KO HT1080 cells (KO) expressing ALCAM-Iso1 (KO + Iso1) or ALCAM-Iso2 (KO + Iso2) treated with 24 h conditioned medium (CM) collected from either HT1080 ALCAM-KO cells (KO CM) or HT1080 KO + Iso2 cells (Iso2 CM). (**b**) Quantification of *in vitro* cell aggregation assay. Clusters were binned into single cell, small (Sm), medium (Med), large (Lrg) or extra-large (XL) clusters based on pixel area. Distribution of clusters across size bins was represented as percent of total area of all clusters. P-values were calculated using Chi-square test for trend and are listed in Supplementary Table ST5. Results are representative of three independent experiments.

In order to determine if elevated ectodomain shedding from ALCAM-Iso2 could antagonize ALCAM-mediated cell-cell adhesion, we analyzed cell aggregation, as described above, in the presence of fresh complete medium (Untreated), conditioned medium from ALCAM-KO cells (KO CM), or conditioned medium from KO + Iso2 cells (Iso2 CM). EGTA was added to inhibit non-specific effects from calcium-dependent adhesion molecules. In the presence of EGTA, KO + Iso1 cells formed significantly larger clusters than KO + Iso2 cells (P = 0.0031, Sup. Table ST5), confirming that this phenotype is ALCAM-specific and Ca^2+^ independent (Fig. 6 *Untreated*). Treatment of KO + Iso1 or KO + Iso2 cells with KO CM did not substantially alter their ability to aggregate, nor did the treatment with Iso2 CM affect aggregation of parental or ALCAM-KO cells (Fig. 6, *KO CM and Iso2 CM*). Conversely, the treatment of KO + Iso1 and KO + Iso2 cells with Iso2 CM greatly reduced the formation of extra-large and large clusters, respectively (Fig. 6, *KO CM vs. Iso2 CM*, P < 0.0001 and P = 0.0015, Sup. Table ST5). In fact, KO + Iso1 cells treated with Iso2 CM formed clusters nearly identical in size to untreated KO + Iso2 cells (P = 0.1013, Sup. Table ST5). Taken together, these data indicate that alternative splicing of *ALCAM* not only diminishes intrinsic adhesion, but that shed ALCAM-Iso2 (ECD-55 and ECD-40) can also disrupt cell-cell adhesion by antagonizing homotypic ALCAM interactions in a paracrine-like manner.

### Isoform-specific differences in cell-cell adhesion are controlled by ectodomain shedding in a bladder cancer cell line

Previously, we have shown that shed ALCAM is detected in the serum and urine of bladder cancer patients at a median level of 74.880 and 2.177 ng/mL, respectively^19^. Our current data suggest that shed ALCAM-Iso2 can antagonize homotypic ALCAM interactions at a concentration of 30 ng/mL (Fig. 3a, Fig. 6). Because shed ALCAM-Iso2 can disrupt cell-cell adhesion at a physiologically relevant concentration and high ALCAM shedding is prognostic of poor patient outcome in bladder cancer^19^, we explored the phenotype of ALCAM-Iso1 and ALCAM-Iso2 in the bladder cancer cell line, UMUC-3. Similar to HT1080 cells, UMUC-3 ALCAM-KO cells (KO) transfected with ALCAM-Iso2 (KO + Iso2) shed ALCAM 2.3-fold more than cells transfected with ALCAM-Iso1 (KO + Iso1, Sup. Fig. S6a). Additionally, by immunoblot, ALCAM-Iso2 is shed equally at two distinct sites in the ECD, while ALCAM-Iso1 is shed primarily at the site corresponding to ADAM17 proteolysis (Sup. Fig. S6b). These data suggest that the differential processing of ALCAM-Iso1 and ALCAM-Iso2 is evident in multiple cancer cell types.

**Figure 7:**
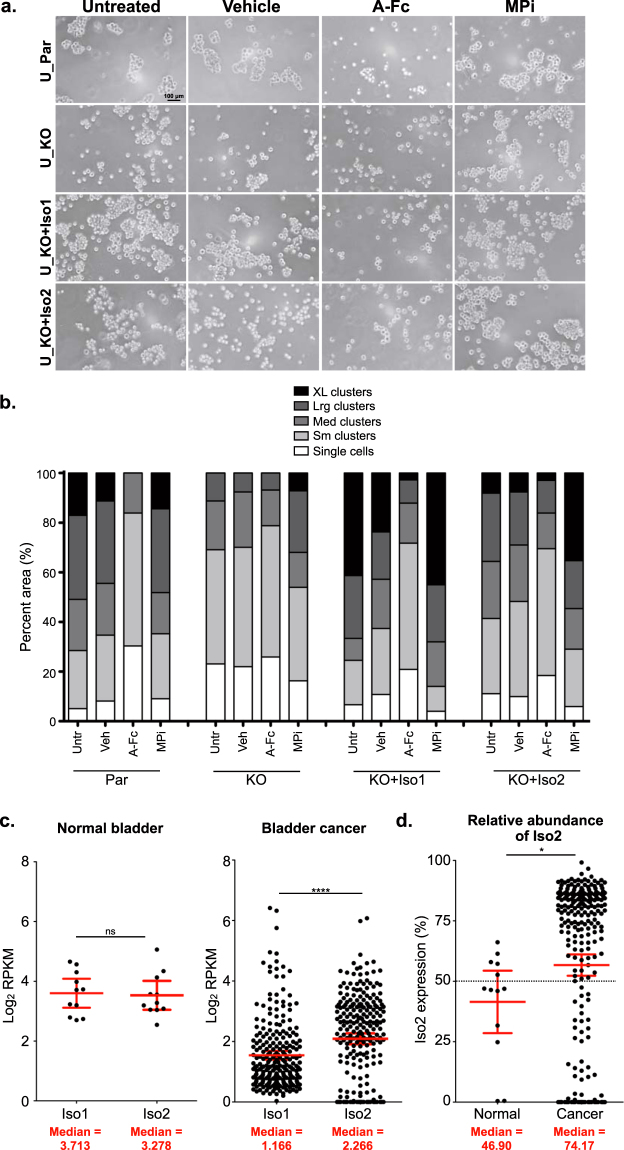
ALCAM-Iso2 expression correlates with disease progression. (**a**) Representative images of *in vitro* cell aggregation analysis of parental UMUC-3 cells (U_Par) and UMUC-3 ALCAM knockout cells (U_KO) transiently expressing ALCAM-Iso1 (U_KO + Iso1) or ALCAM-Iso2 (U_KO + Iso2). Veh, vehicle (0.4% DMSO), A-Fc, soluble ALCAM-Fc (10 μg/mL), MPi, metalloprotease inhibitor (GM6001, 10 μM). (**b**) Quantification of *in vitro* cell aggregation assay. Clusters were binned into single cell, small (Sm), medium (Med), large (Lrg), or extra-large (XL) clusters based on pixel area. Distribution of clusters across size bins was represented as percent of total area of all clusters. P-values were calculated using Chi-square test for trend and are listed in Supplementary Table ST6. Results are representative of three independent experiments. (**c**) Expression of ALCAM-Iso1 and ALCAM-Iso2 in normal bladder and bladder cancer. Data for normal bladder tissue were extracted from the GTEx Portal. Data for bladder cancer tissue were extracted from the TCGA bladder cancer cohort. p-values were calculated using paired t-test, ****p < 0.0001. Graphs display mean with 95% confidence interval. Medians are reported. (**d**) Relative abundance of ALCAM-Iso2 in normal and bladder cancer tissue. P-value calculated with Mann-Whitney U test. Graphs display mean with 95% confidence interval. Medians are reported.

We then used parental UMUC-3 transiently transfected with control vector (U_Par), ALCAM-KO UMUC-3 cells transiently transfected with control vector (U_KO), ALCAM-Iso1 (U_KO + Iso1), or ALCAM-Iso2 (U_KO + Iso2) to verify that alternative splicing of *ALCAM* confers differential susceptibility to proteolysis and controls cell-cell adhesion. U_Par cells formed cell clusters of varying sizes with 17% extra-large clusters, 34% large clusters, 21% medium clusters, 23% small clusters, and 5% single cells, while U_KO cells formed significantly smaller clusters, with single cells and small clusters accounting for 69% of the cells and medium and large clusters accounting for 31% of cells (Fig. 7a,b, *Untreated*, p < 0.0001, Sup. Table ST6). Rescue with ALCAM-Iso1 (U_KO + Iso1) promoted cell clusters larger than that of parental cells with extra-large clusters accounting for 41% of cells and large clusters accounting for 25% of cells (Fig. 7a,b, P = 0.0113, Sup. Table ST6). Meanwhile, cells with ALCAM-Iso2 rescue (U_KO + Iso2) formed clusters significantly smaller than both U_Par and U_KO + Iso1 cells (P = 0.0094, P < 0.0001, respectively, Sup. Table ST6) with 41% small clusters and single cells and only 35% large and extra-large clusters (Fig. 7a,b, *Untreated*).

Consistent with our previous findings (Fig. 5), soluble ALCAM-Fc disrupted ALCAM-ALCAM interactions in all groups except KO cells, leading to significantly smaller cell clusters (Fig. 7a,b, *A-Fc*; p < 0.0001, Sup. Table ST6). In U_Par cells, small clusters and single cells accounted for 83% of the cell distribution after ALCAM-Fc treatment, while ALCAM-Fc treatment in U_KO + Iso1 and U_KO + Iso2 resulted in 70% and 69% small clusters and single cells, respectively (Fig. 7a,b). Finally, treatment with GM6001 significantly increased the size of cell clusters in U_KO + Iso2 cells, increasing large and extra-large clusters to 54% (Fig. 7a,b, *MPi*; P = 0.0003, Sup. Table ST6). Again, these data suggest that the effects of alternative splicing of ALCAM on cell-cell cohesion persist across multiple cancer cell types.

### Changes in *ALCAM* isoform expression correlate with disease progression in bladder cancer

Together, these data indicate that differences in *ALCAM* isoform expression can be important for bladder cancer progression. To investigate this further, we evaluated *ALCAM* expression in both normal bladder and bladder cancer tissue. Using the publicly available Genotype-Tissue Expression (GTEx Portal)^40^ dataset, we quantified ALCAM-Iso1 and ALCAM-Iso2 expression in normal bladder and showed that ALCAM-Iso1 and ALCAM-Iso2 are expressed in relatively equal levels (Fig. 7c, left panel). Additionally, using The Cancer Genome Atlas (TCGA) urothelial bladder carcinoma cohort^41^, we quantified *ALCAM* isoform expression in human bladder tumors and showed that, ALCAM-Iso2 is expressed two-fold higher than ALCAM-Iso1 (P < 0.0001, Fig. 7c, right panel). These data indicate that there is a switch to ALCAM-Iso2 dominated expression in bladder cancer, compared to normal tissue. This was validated by directly comparing the relative abundance of ALCAM-Iso2 between the GTEx (normal bladder) and TCGA (bladder cancer) cohorts (P = 0.0224, Fig. 7d). This clinical data supports our mechanistic data and suggests that ALCAM-Iso1 expression promotes high cell-cell cohesion, while a switch to ALCAM-Iso2 dominated expression promotes lower cell-cell adhesion, a permissive environment for single cell dispersion, and, as a result, enhanced migration and metastasis. Thus, we suggest that differential splice isoform expression provides an explanation for increased ALCAM shedding in patients with cancer and provides a mechanism by which ALCAM detected in biofluids is prognostic of poor outcome.

## Discussion

The overarching goal of this study was to determine how ALCAM shedding regulates tumor cell biology. In this manuscript, we describe how alternative splicing of *ALCAM* controls the functional contribution of ALCAM to tumor biology.

Dynamic alteration of cell adhesion is an integral step to cancer progression^1,42^. While many adhesion molecules have been shown to control cancer progression, the mechanisms underlying dynamic regulation of these molecules remain unclear. Unlike many of the classic oncogenes and tumor suppressors, the cell adhesion molecules that control malignant progression are rarely genetically mutated, amplified, or deleted. Rather than genomic alterations, the contributions of these adhesion molecules appear to be controlled primarily at the transcriptional and post-transcriptional level. The function of many Ig-CAMs, including ALCAM, is regulated through proteolytic shedding of the ectodomain from the cell surface^43^. ALCAM’s participation in malignant progression has been recognized in numerous studies^9,17,18,20–24^ and we recently linked elevated ALCAM shedding directly to poor patient outcome^19,44^. However, the mechanism controlling proteolytic shedding of ALCAM was unknown.

In this study, we demonstrate that alternative splicing is responsible for controlling the susceptibility of ALCAM to proteolytic shedding. Loss of exon 13 in ALCAM-Iso2 allows for MMP14-dependent proteolytic cleavage at a membrane-distal site which leads to a 10-fold increase in ALCAM-Iso2 shedding compared to the canonical isoform, ALCAM-Iso1 (Fig. 3). The role of metalloproteases in cancer progression is still being defined and clinical attempts to target MMP activity have failed^45^. However, studies such as ours help define the contribution of specific cleavage events to disease progression. Since many proteases are promiscuous and target several substrates^46,47^, changing proteolytic susceptibility through alternative splicing allows for specific and dynamic control of ALCAM proteolysis. Therefore, differential expression of ALCAM-Iso1 and ALCAM-Iso2, as opposed to altering protease activity, allows for intrinsic control of ALCAM shedding and adhesion function without affecting other targets of ADAM17 or other metalloproteases.

We originally hypothesized that exon 13 might contain the ADAM17 cleavage site and that the splicing would therefore remove proteolytic susceptibility to ADAM17 in a manner similar to what has been reported for Cell Adhesion Molecule 1 (CADM1) and the epithelial growth factor receptor family member (ErbB4)^48,49^. However, we discovered that the loss of exon 13 by splicing is unique in the fact that it makes ALCAM more susceptible to proteolytic cleavage in a separate, membrane-distal site, outside of exon 13. Moreover, this proteolytic event is MMP14-dependent and distinct from ADAM17 activity. Thus, the ALCAM ectodomain appears to contain a second proteolytic site that is exposed or unmasked upon alternative splicing of exon 13. To our knowledge, this is the first observation of a masked proteolytic site (within the canonical sequence) that is revealed upon the alternative splicing of an adjacent exon.

The specific regulation of ALCAM function through differential expression of *ALCAM* isoforms has implications in various biological processes. ALCAM has known roles in non-pathologic processes requiring static and stable cell adhesion such as T-cell activation and maintenance of the blood-brain barrier^15,50,51^, as well as roles in dynamic processes such as transendothelial migration and neuronal guidance^14,16^. To this point, the vast majority of ALCAM function has been investigated using the canonical isoform, ALCAM-Iso1. We hypothesize that dynamic regulation of cell adhesion through differential expression of *ALCAM* isoforms would be vital to all ALCAM-mediated functions, both pathological and non-pathological. Our results clearly indicate that the role of ALCAM in many physiological processes may be more complicated and more dynamic than first indicated.

While broadly applicable in the field of cell adhesion, our findings highlight the importance of regulating ALCAM shedding in cancer. In our direct comparison of ALCAM-Iso1 and -Iso2, we show that expression of ALCAM-Iso2 increases ectodomain shedding up to ten-fold (Fig. 3a, Sup. Fig. S6a), which diminishes cell-cell adhesion (Figs. 5, 6 and 7). Additionally, ALCAM-Iso2 expression mediated an increase in metastasis and tumor cell dissemination (Figs. 1f and 2g). Finally, we show a switch from nearly equal isoform expression in normal bladder tissue to ALCAM-Iso2-dominated expression in bladder cancer (Fig. 7c,d), which further supports the role of ALCAM-Iso2 in tumor progression. While the regulation of alternative splicing has not been studied for *ALCAM* specifically, aberrations in alternative splicing in cancer can be attributed to several factors such as genetic abnormalities in spliceosome machinery or, more commonly, to dysregulation of expression and localization of *trans*-acting splicing factors^52^. Therapeutic targeting of alternative splicing in cancer is an active field of research; therefore, identifying the regulatory mechanisms guiding alternative splicing in *ALCAM* could potentially lead to novel cancer therapies. Additionally, given our previous work that indicates that high ALCAM shedding is predictive of poor patient outcome^19,44^, we hypothesize that elevated ALCAM-Iso2 expression may be the cause of increased ALCAM shedding in patients with aggressive disease. This combination of experimental and clinical observations warrants future investigations into the value of ALCAM-Iso2 tissue expression as a predictive and prognostic biomarker.

Although this is the first study to attribute differential proteolysis of ALCAM to alternative splicing, smaller soluble forms of ALCAM generated by proteolysis of the ECD have been observed in other studies. Fabbi and colleagues observed the generation of two soluble ALCAM fragments (95 kDa and 65 kDa (glycosylated)) and attributed the generation of both fragments to ADAM17 activity in neuroblastoma cell lines^53^. Bongarzone and colleagues also observed the generation of soluble ALCAM fragments at 96 kDa and 60 kDa (glycosylated) or 55 kDa and 35 kDa (deglycosylated) in thyroid cancer cell lines^39^. These soluble forms align well with the soluble forms we observed from the proteolysis of ALCAM-Iso2 and may indicate that the cell lines used in these reports express ALCAM-Iso2. The findings we report here enable further interpretation of the existing literature on ALCAM and its function.

Our work also provides an opportunity to direct further research into the biology of ALCAM and the cell-adhesion it controls. Understanding how the alternative splicing is regulated and how this alternative splicing unmasks an MMP14-sensitive membrane-distal cleavage site in ALCAM-Iso2 will provide further insight into the intrinsic regulatory mechanisms that control the physiology and pathology of multi-cellular adhesions. Further structure-function studies of this masked proteolytic site can reveal dynamic regulatory systems intrinsic to normal physiology that are coopted by cancer to provide this disease a dynamic and tunable mechanism to control migration, dissemination, and metastasis. In cancer, detecting the ALCAM shedding, as well as the switch from ALCAM-Iso1 to ALCAM-Iso2 may function as an indicator of disease progression. Additionally, it may be possible to target this regulatory process with a therapeutic intervention that can benefit cancer patients as well as individuals with other cell adhesion-related pathologies.

**Figure 8:**
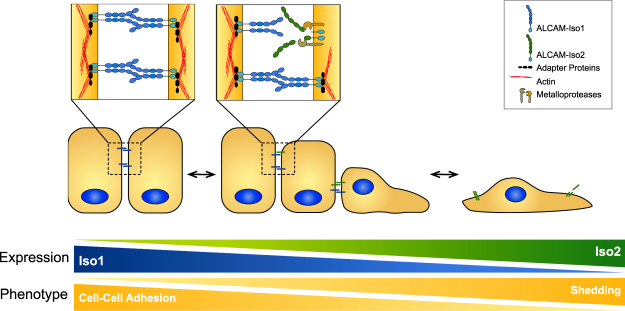
Expression of alternative *ALCAM* splice isoforms modulates cell-cell adhesion through differential susceptibility to proteolysis. Our data demonstrate that alternative splicing of *ALCAM* can alter cell-cell adhesion through differential susceptibility to proteolysis of the extracellular domain. Cells expressing ALCAM-Iso2 are more likely to undergo single cell dispersion and, therefore, metastasize more frequently from the primary tumor.

In conclusion, we have shown that alternative splicing of *ALCAM* can specifically and dynamically regulate ALCAM-mediated cell-cell adhesion by introducing a proteolytic susceptibility adjacent to the spliced exon (Fig. 8). The increased shedding of ALCAM-Iso2 leads to reduced cell-cell adhesion *in vitro* and provides a mechanism by which ALCAM-Iso2 can promote metastasis *in vivo*. The elevated expression of *ALCAM*-Iso2 observed in the tumor tissue from bladder cancer patients (Fig. 7c,d) gives further support to the hypothesis that alternative splicing of ALCAM contributes to the progression of cancer. Given our previous work, which indicates that high ALCAM shedding is predictive of poor patient outcome^19,44^, we hypothesize that elevated ALCAM-Iso2 expression may be the cause of increased ALCAM shedding in patients with aggressive disease. This study is the first evaluation of biochemical and functional differences between ALCAM-Iso1 and ALCAM-Iso2, and our findings warrant further investigation into the role of alternative splicing of *ALCAM* in both normal and pathological processes.

## Materials and Methods

### Cell Culture, Plasmids, Transfections, and Inhibitors

HT1080 (fibrosarcoma) and UMUC-3 (transitional cell carcinoma) cells were obtained from the ATCC (CCL-121 and CRL-1749, respectively). Cell lines were maintained in RPMI (HT1080) or DMEM (UMUC-3) supplemented with penicillin/streptomycin, sodium pyruvate, nonessential amino acids and 10% fetal bovine serum and cultured at 37 °C with 5% CO_2_. Knockout cell lines were generated using a CRISPR/Cas9 gRNA targeting the signal peptide of ALCAM (AGACGGTGGCGGAGATCAAG, Horizon Discovery, St. Louis, Missouri). The level of protein expression was verified by flow cytometry for cell surface ALCAM and by immunoblot for total ALCAM expression in whole cell lysates. HT1080 isoform variants were stably transduced with lentiviral particles to express the ALCAM isoform indicated as well as a labeling vector containing firefly luciferase with either mTagCerulean or mTagRFP2 fluorescent protein (Sup. Fig. S2a). Viral plasmids were constructed using MultiSite Gateway (ThermoFisher Scientific, Waltham, Massachusetts) on the pLenti6.2/V5-DEST backbone. Viral particles were produced in LentiX 293 T cells (Clontech, Mountainview, California). Transductions were performed with 5 μg/mL polybrene. Transient transfections were performed using X-tremeGENE HP (Sigma Aldrich, St. Louis, Missouri) with 2 μg plasmid DNA, or 800 ng siRNA (Mission esiRNA human MMP14, Sigma Aldrich) as indicated. Protease inhibitors are listed in Supplementary Table ST2.

### Fluorescence Activated Cell Sorting (FACS) and Flow Cytometry

Following transduction described above, cell populations were purified by FACS. Cells were stained for cell surface ALCAM for 30 min (0.8 μg/10^6^ cells, anti-ALCAM (extracellular domain), MAB6561, R&D Systems, Minneapolis, Minnesota). Primary antibody binding was detected with Alexa647-conjugated anti-mouse IgG (0.25 μg/10^6^ cells, A28181, ThermoFisher Scientific, Waltham, Massachusetts). Cells were sorted for either the RFP + /GFP + /ALCAM + or the Cerulean + /GFP + /ALCAM + population, as appropriate. To quantify the amount of cell surface ALCAM and verify success of FACS, cells were stained as described above and analyzed by flow cytometry.

### Avian Embryo Metastasis Models

Spontaneous and experimental metastasis experiments were performed as described previously^34,36^. In accordance with the Public Health Services policy on Humane Care and Use of Laboratory Animals, Vanderbilt Institutional Animal Care and Use Committee (IACUC) has determined that avian embryos developmental day 17 and younger are not considered vertebrate animals and therefore do not require specific protocol approval. Vanderbilt IACUC has reviewed and approved the following experimental and euthanasia procedures.

#### Spontaneous Metastasis (Xenograft)

Fertilized chicken eggs were placed in 37 °C incubator at 55% relative humidity on day 1 post-fertilization. The chorioallantoic membrane (CAM) of day 10 embryos was dropped away from the eggshell by displacing the air cell. Cells were dissociated, counted, and resuspended in sterile 0.9% saline at 20 × 10^6^ cells/mL. The dropped CAM was damaged slightly with sterile cotton tipped swabs. 25 μL of cell suspension (500,000 cells) were placed on the dropped CAM and eggs were returned to the incubator. After 5 days of growth, embryos were euthanized by decapitation, and xenografts were harvested, weighed, and fixed in 10% zinc formalin. Fixed tumors were paraffin embedded, sectioned, and stained with hemotoxylin and eosin to visualize local invasion. To quantify metastasis to the distal CAM, the eggshell was cut in half along the long axis, chicks were dissected from the shell, and sections of the distal CAM were dissected using a cork borer. Dissected CAM tissue was incubated with D-luciferin (600 μg/mL) for 45 minutes and luciferase activity was quantified using a digital gel documentation system (G:Box; Syngene, Frederick, Maryland) as a measure of cell number.

#### Experimental Metastasis (Intravenous)

Fertilized chicken eggs were placed in 37 °C incubator at 55% relative humidity on day 1 post-fertilization. Day 11 embryos were candled to locate the allantoic vein and the direction of blood flow. Cells were dissociated, counted, and resuspended at 0.25 × 10^6^ cells/mL in sterile 0.9% saline. A window was cut into the eggshell at the injection site using a Dremel™ tool (Racine, Wisconsin). 100 μL of cell suspension (25,000 cells) were injected intravenously using an insulin syringe. Eggs were returned to the incubator for 5 days. Upon harvest, embryos were euthanized by decapitation, and metastasis was quantified by luciferase activity as described above.

#### *Ex Vivo* Imaging of Tumor Colonies

To analyze colony morphology, we performed the experimental metastasis assay as described above. However, upon harvest at 6 days post-injection, eggs were cut in half along the long axis, chicks were dissected from the shell, euthanized by decapitation, and three sections of CAM were dissected using a cork borer. Colonies were imaged using an upright fluorescent microscope (BX-61 WI, Olympus, Pittsburgh, Pennsylvania) and Volocity Imaging Software (Perkin Elmer, Waltham, Massachusetts). Colony size was determined using a custom KNIME^54^ workflow (Zurich, Switzerland). In brief, images were thresholded and segmented, after which object size, reflective of colony size and morphology, was determined for each colony. All images were processed with the same workflow. Objects were assigned to one of four bins by size including single cells (150–1,000 pixels), small colonies (1,001–20,000 pixels), medium colonies (20,001–100,000 pixels), and large colonies (>100,000 pixels). Data are presented as percent of total area for each bin category.

### Detecting ALCAM Shedding

Cells were plated in 6-well plates at 80% confluency and allowed to adhere overnight. Complete medium was removed and cells were incubated with indicated protease inhibitors or vehicle in serum-free medium for 24 h (Sup. Table ST2). Conditioned medium was collected, spun at 500 g for 5 min to remove cell debris, and half of the sample was concentrated 25 × by acetone precipitation. Cells were lysed using 1% Triton X-100 lysis buffer for 30 min on ice. Lysates were clarified by centrifugation at 14,000 g for 15 min at 4 °C. Following deglycosylation with PNGase-F (New England Biolabs, Ipswitch, MA), ALCAM protein expression (whole cell lysate) and shedding (conditioned medium) were detected by immunoblot with anti-ALCAM (intracellular domain; 1G3A)^44^ and anti-ALCAM (extracellular domain; MOG/07, Leica Biosystems, Buffalo Grove, Illinois), respectively. ALCAM protein expression and shedding were quantified using human ALCAM DuoSet ELISA (R&D Systems, Minneapolis, Minnesota) from un-concentrated whole cell lysate and conditioned medium, respectively.

### Adhesion to Immobilized ALCAM-Fc

A 96-well high-binding plate was coated with protein G (2 μg/mL in 100 mM bicarbonate/carbonate buffer, pH 9.2) overnight at 4 °C. The plate was washed with phosphate buffered saline (PBS) and coated overnight at 4 °C with recombinant Fc-tagged ALCAM extracellular domain (ALCAM-Fc, 1 μg/mL) in appropriate wells; negative control wells were not coated with ALCAM-Fc. The plate was washed with PBS and positive control wells (equated to 100% binding) were then coated with Collagen Type 1 (100 μg/mL) overnight at 4 °C. The plate was washed with PBS and 2.5 × 10^4^ cells/well were added and incubated at 37 °C and 5% CO_2_ for 4 hours. Non-adherent cells were washed away by gentle pipetting and aspiration. Cells were fixed with 4% paraformaldehyde (4% w/vol) and stained with crystal violet (0.5% vol/vol in 20% methanol). Stain was extracted with acetic acid (20% vol/vol). Absorbance was read on automated plate reader (BioTek, Winooski, VT). Cell adhesion was quantified as percent of adhesion compared to wells coated with collagen type I coated (considered 100% adhesion).

### *In vitro* Cell Aggregation

Cells were detached using non-enzymatic cell dissociation buffer and plated in 24-well low-attachment plates at 2.5 × 10^4^ cells/well. For conditioned medium experiments, EGTA was added to a final concentration of 1 mM to inhibit calcium-dependent cell adhesion. Cells were incubated with indicated treatments for 12 hours and cell clusters were imaged using a light microscope (TMS-F, Nikon, Tokyo, Japan) fitted with a D90 SLR camera (Nikon, Tokyo, Japan). Ten images were captured for each experimental group and analyzed in FIJI (ImageJ)^55^ by segmenting with Weka Trainable Segmentation^56^ and calculating cluster size with the Particle Analysis plug-in. Clusters were assigned to one of four bin categories by size including single cells (200–400 pixels), small colonies (401–2,000 pixels), medium colonies (2,001–4,000 pixels), large colonies (4,001–10,000 pixels), and extra-large (>10,000 pixels). Data are presented as percent of total area for each bin category.

### Isoform Expression in Human Tissue

GTEx Transcript reads per kilobase per million mapped reads (RPKMs) were downloaded from the GTEx Portal^40^ (Analysis version 6, normal bladder tissue), and transcripts ENST00000306107.5 (ALCAM-Iso1) and ENST00000306107.5 (ALCAM-Iso2) were analyzed. For TCGA (urothelial bladder carcinoma)^41^, per quantile normalized RPKMs for bladder cancer we downloaded from the GDC portal (https://portal.gdc.cancer.gov), and transcripts ENST00000306107.5 (ALCAM-Iso1) and ENST00000306107.5 (ALCAM-Iso2) were analyzed. RPKM values were transformed using Log_2_(x + 1) for ALCAM-Iso1 and -Iso2 and reported for normal bladder (GTEx Portal) and bladder cancer (TCGA) datasets. In addition, the relative abundance of ALCAM-Iso2 was calculated for each patient and used to compare isoform expression in normal bladder compared to bladder cancer.

### Statistical analysis

All statistical analyses were performed using Prism 5 for Mac OS X (GraphPad Software, Inc, La Jolla, California). For statistical analysis of xenograft weight, metastasis to distal CAM, ELISA quantifications, and colony size, experimental groups were compared to control groups using Kruskal-Wallis test with Dunn’s post-test. Chi-square test for trend was used to determine statistically significant differences in size distribution between two groups for colony sizes and aggregation assays. All pair-wise Chi-square test for trends are reported in Supplementary Tables ST3, ST4 and ST5. ALCAM-Iso1 expression was compared to ALCAM-Iso2 expression in both normal bladder and bladder cancer using paired t-test on Log_2_(x + 1) transformed data, while Mann-Whitney U was used to compare differences in relative abundance of ALCAM-Iso2 between normal bladder and bladder cancer.

### Data availability

The datasets analyzed in this manuscript are publicly available using the GTEx Portal (https://www.gtexportal.org/home/) and The Cancer Genome Atlas (TCGA) urothelial bladder carcinoma cohort (https://cancergenome.nih.gov/cancersselected/UrothelialBladderCarcinoma). ALCAM isoform expression was extracted for transcript accession codes ENST00000306107.5 (ALCAM-Iso1) and ENST00000306107.5 (ALCAM-Iso2).

## Electronic supplementary material

Supplementary Material
