# Supplementary material for: Alternative splicing of ALCAM enables tunable regulation of cell-cell adhesion through differential proteolysis
Source: Sci Rep. 2018 Feb 16;8:3208. doi: 10.1038/s41598-018-21467-x (PMC5816644; doi:10.1038/s41598-018-21467-x)
Supplement: Supplementary file 1 — Supplementary Material [file 41598_2018_21467_MOESM1_ESM.pdf]

Alternative splicing of *ALCAM* enables tunable regulation of cell-cell adhesion through differential proteolysis

Katie E. Hebron[1], Elizabeth Y. Li[2], Shanna A. Arnold Egloff[3,4], Ariana K. von Lersner[1], Chase Taylor[3,4], Joep Houkes[5], David K. Flaherty[6], Adel Eskaros[3], Thomas P. Stricker[3], Andries Zijlstra[1,3]*

[1] Vanderbilt University Medical Center, Program in Cancer Biology
[2] Massachusetts Institute of Technology, Department of Biology
[3] Vanderbilt University Medical Center, Department of Pathology, Microbiology and Immunology
[4] Department of Veterans Affairs, Tennessee Valley Healthcare System
[5] Wageningen University and Research, Department of Microbiology
[6] Vanderbilt University Medical Center, Vanderbilt Vaccine Center

*Corresponding Author:
Andries Zijlstra
Vanderbilt University Medical Center
1161 21st Ave S.
C-2102B Medical Center North
Nashville, TN 37232
Andries.Zijlstra@Vanderbilt.Edu

SUPPLEMENTARY MATERIAL

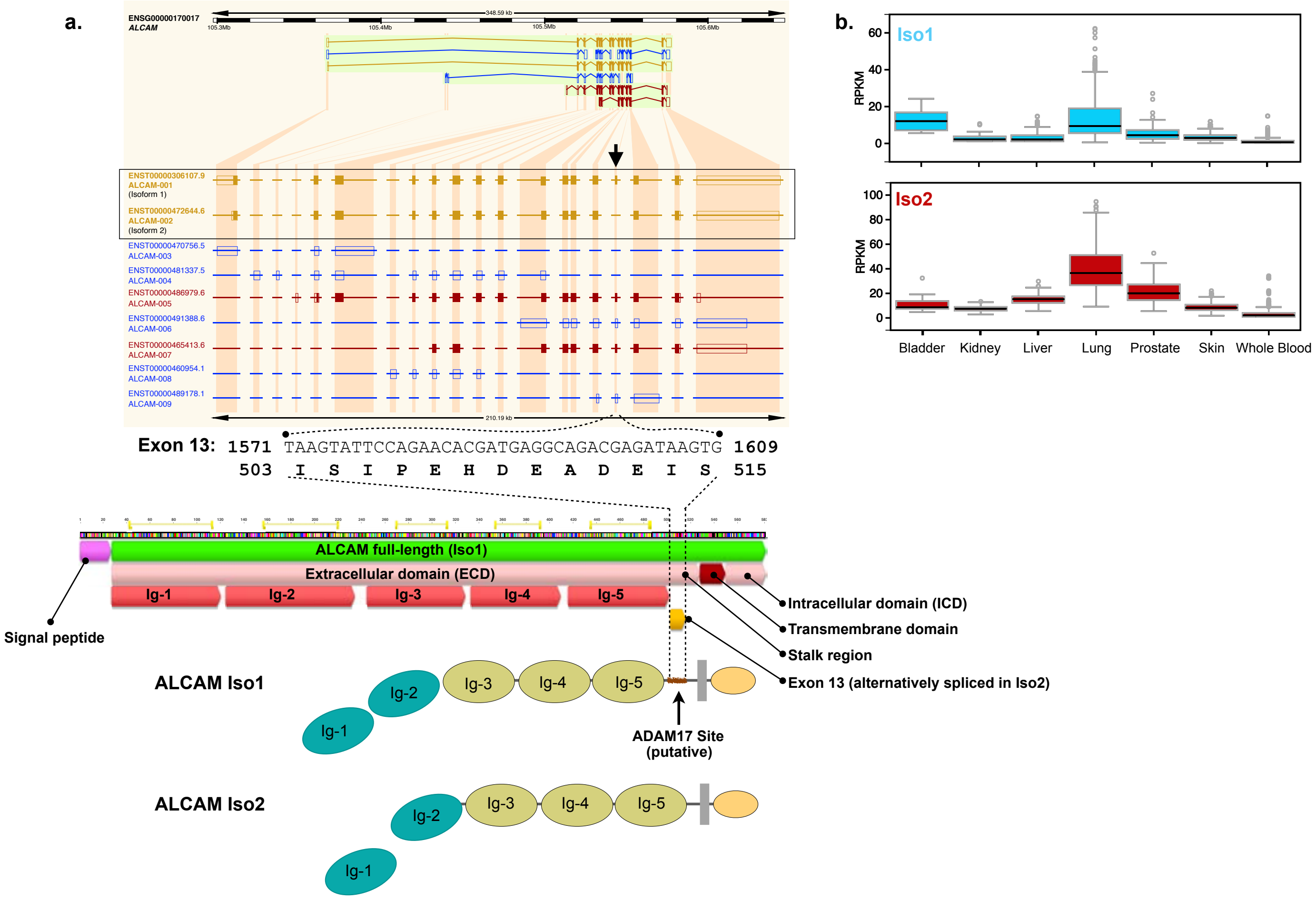

Supplementary Figure S1. ALCAM is expressed as two distinct splice isoforms in normal human tissue. (**a**) Reported potential splice isoforms of ALCAM. Black arrow indicates exon 13. Gold indicates protein coding transcripts with identical annotations in both Ensembl and HAVANA systems. Red indicates protein coding transcripts with non-identical annotations in Ensembl and HAVANA systems. Blue indicates non-coding transcript. Schematic depicts protein structure of ALCAM-Iso1 and ALCAM-Iso2. (**b**) Isoform expression in indicated normal human tissue. Data extracted from GTEx Portal.

a.

	Parental HT1080			ALCAM-knockout HT1080		
**Name**	**Parental**	**Iso1-OE**	**Iso2-OE**	**KO**	**KO+Iso1**	**KO+Iso2**
**ALCAM variant**	-	Iso1	Iso2	-	Iso1	Iso2
**CRISPR KO**	No	No	No	Yes	Yes	Yes
**pRBow label**	RFP	Cerulean	RFP	RFP	RFP	RFP

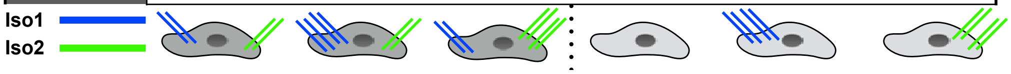

b.

Parental HT1080

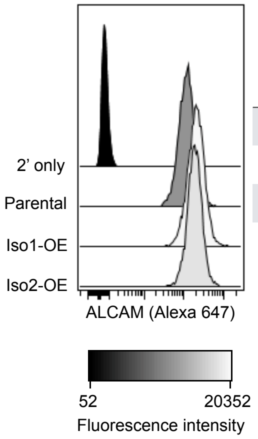

Sample	Median intensity
2' only	52
Parental	14852
Iso1-OE	20352
Iso2-OE	18658

ALCAM-knockout HT1080

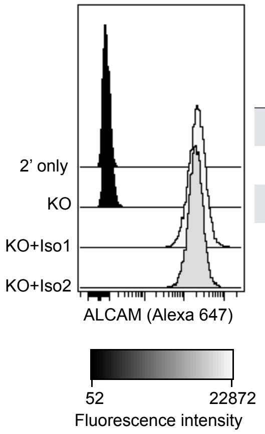

Sample	Median intensity
2' only	52
KO	108
KO+Iso1	22872
KO+Iso2	20431

Supplementary Figure S2. Stable cell line description. (**a**) Table describing each of the main stable cell lines used. (**b**) Surface ALCAM expression of cells described in (a), as determined by flow cytometry.

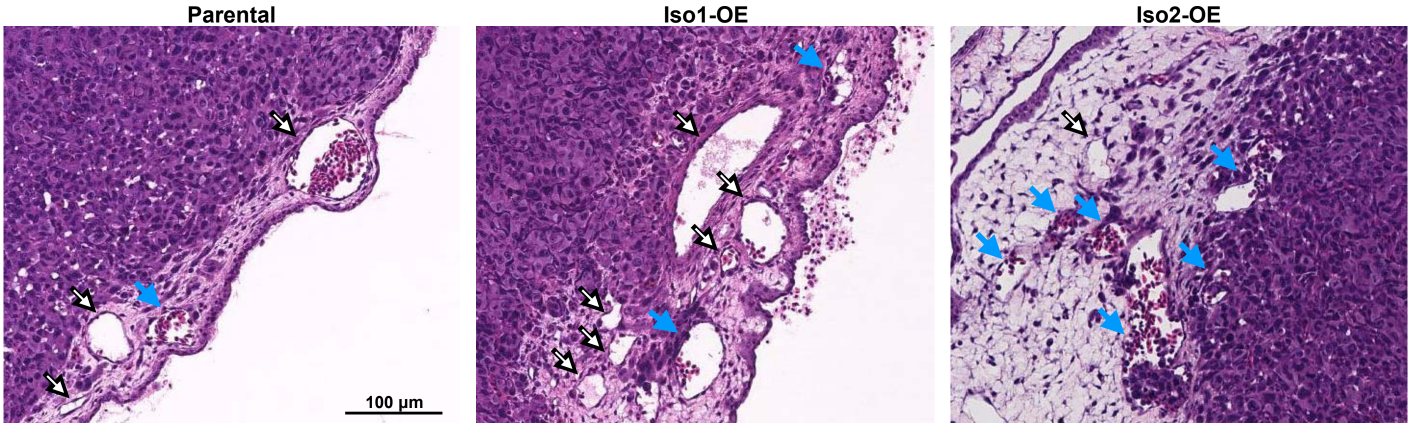

Supplementary Figure S3: ALCAM-Iso2 cells show higher incidence of intravascular invasion at the primary site. Hematoxylin and Eosin (H&E) stained tumor sections from xenografts of parental HT1080 cells overexpressing ALCAM-Iso1 (Iso1-OE) or ALCAM-Iso2 (Iso2-OE). Tumor adjacent blood vessels were scored positive (➔) or negative (⇨) for intravascular invasion by a blinded pathologist.

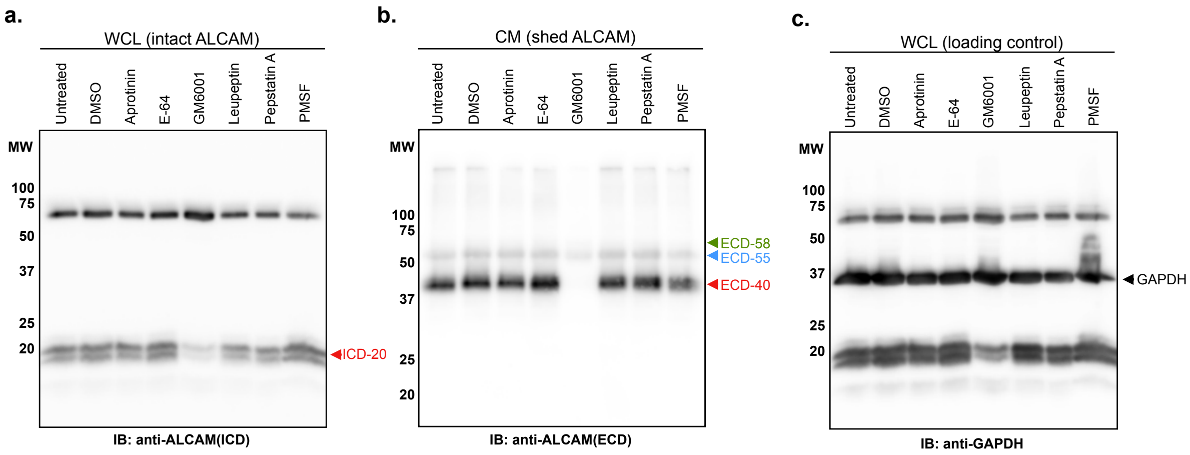

b'

Ratio CM:WCL

1.00	0.96	1.05	0.98	0.08	1.12	1.32	1.01

Supplementary Figure S4. ALCAM-Iso2 shedding is dependent on metalloprotease activity. Immunoblot analysis of extracellular domain shedding of ALCAM-KO HT1080 cells expressing ALCAM-Iso2 (KO+Iso2) treated with various protease inhibitors (listed in Supplementary Table ST2). Results are representative of three independent experiments. (**a**) whole cell lysate (WCL), (**b**) conditioned medium (CM), (**b'**) Changes in shedding were quantified as a ratio between intact ALCAM (WCL), and shed ALCAM (CM), with the untreated control normalized to 1. Samples were derived from the same experiment. Gels and blots were processed in parallel, (**c**) WCL GAPDH loading control, (**a, b, c**) 5 min exposures are shown. Individual blots are outlined in black. WCL blots were redeveloped with HRP-tagged anti-GAPDH for loading control. ICD: intracellular domain, ECD: extracellular domain. ALCAM fragments generated by shedding are marked as follows: 20 kDa intracellular domain fragment (ICD-20, ◀), 58 kDa extracellular domain fragment (ECD-58, ◀), 55 kDa extracellular domain fragment (ECD-55, ◀), and 40 kDa extracellular domain fragment (ECD-40, ◀).

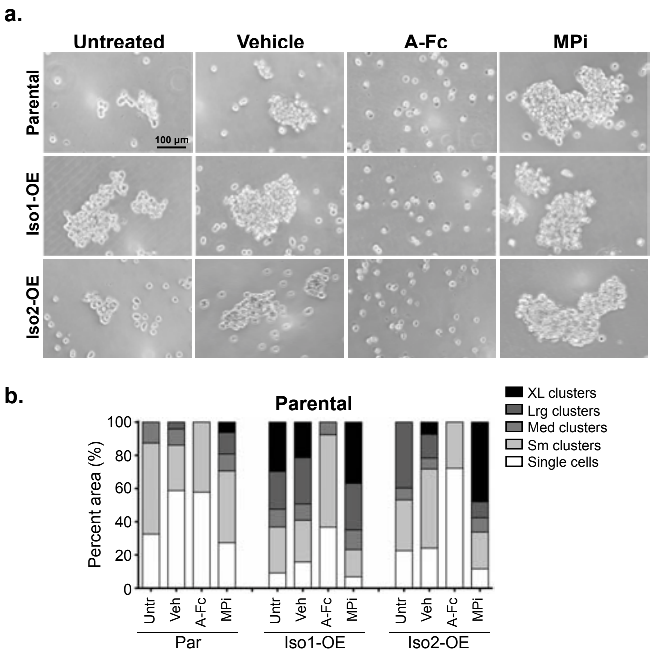

Supplementary Figure S5. ALCAM-Iso1 promotes cell:cell adhesion through intercellular homotypic ALCAM interactions. (**a**) Representative images of *in vitro* cell aggregation analysis of parental HT1080 cells overexpressing ALCAM-Iso1 (Iso1-OE) or ALCAM-Iso2 (Iso2-OE). Veh: vehicle (DMSO), 0.4%; A-Fc: ALCAM-Fc, 10 µg/mL; MPi: metalloprotease inhibitor (GM6001), 10 µM. (**b**) Quantification of *in vitro* cell aggregation assay. Clusters were binned into single cell, small, medium, large, or extra-large clusters based on pixel area. Distribution of clusters across size bins was represented as percent of total area of all clusters. P-values were calculated using Chi-squared test for trend and are listed in Supplementary Table ST4. Results are representative of three independent experiments.

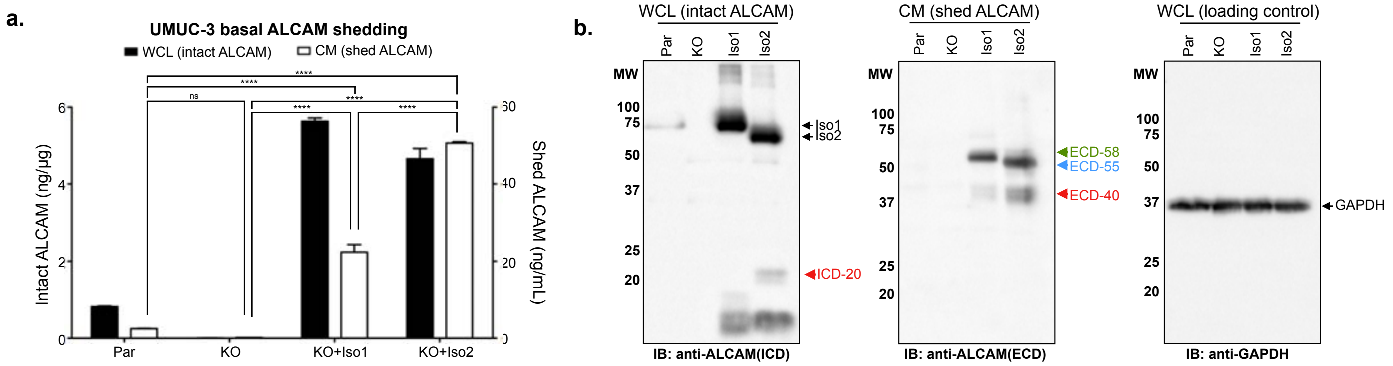

Supplementary Figure S6. Alternative splicing of ALCAM leads to enhanced proteolytic susceptibility in the bladder cancer cell line UMUC-3. (**a**) ALCAM ELISA for quantification of basal extracellular domain shedding in parental UMUC-3 cells (U_Par) and UMUC-3 ALCAM knockout cells (U_KO) transiently expressing ALCAM-Iso1 (U_KO+Iso1) or ALCAM-Iso2 (U_KO+Iso2). WCL: whole cell lysate, CM: conditioned medium. P-values were calculated using Kruskal-Wallis test with Dunn's post-test, ns=not significant, ****P<0.0001. (**b**) Immunoblot analysis of basal extracellular domain shedding of ALCAM-Iso1 and ALCAM-Iso2 in parental UMUC-3 cells (U_Par) and UMUC-3 ALCAM knockout cells (U_KO) transiently expressing ALCAM-Iso1 (U_KO+Iso1) or ALCAM-Iso2 (U_KO+Iso2). ICD: intracellular domain, ECD: extracellular domain. ALCAM fragments generated by shedding are marked as follows: 20 kDa intracellular domain fragment (ICD-20, ◀), 58 kDa extracellular domain fragment (ECD-58, ◀), 55 kDa extracellular domain fragment (ECD-55, ◀), and 40 kDa extracellular domain fragment (ECD-40, ◀). 5 min exposures are shown. Individual blots are outlined in black. WCL blots were redeveloped with HRP-tagged anti-GAPDH for loading control. Full-length blots are shown.

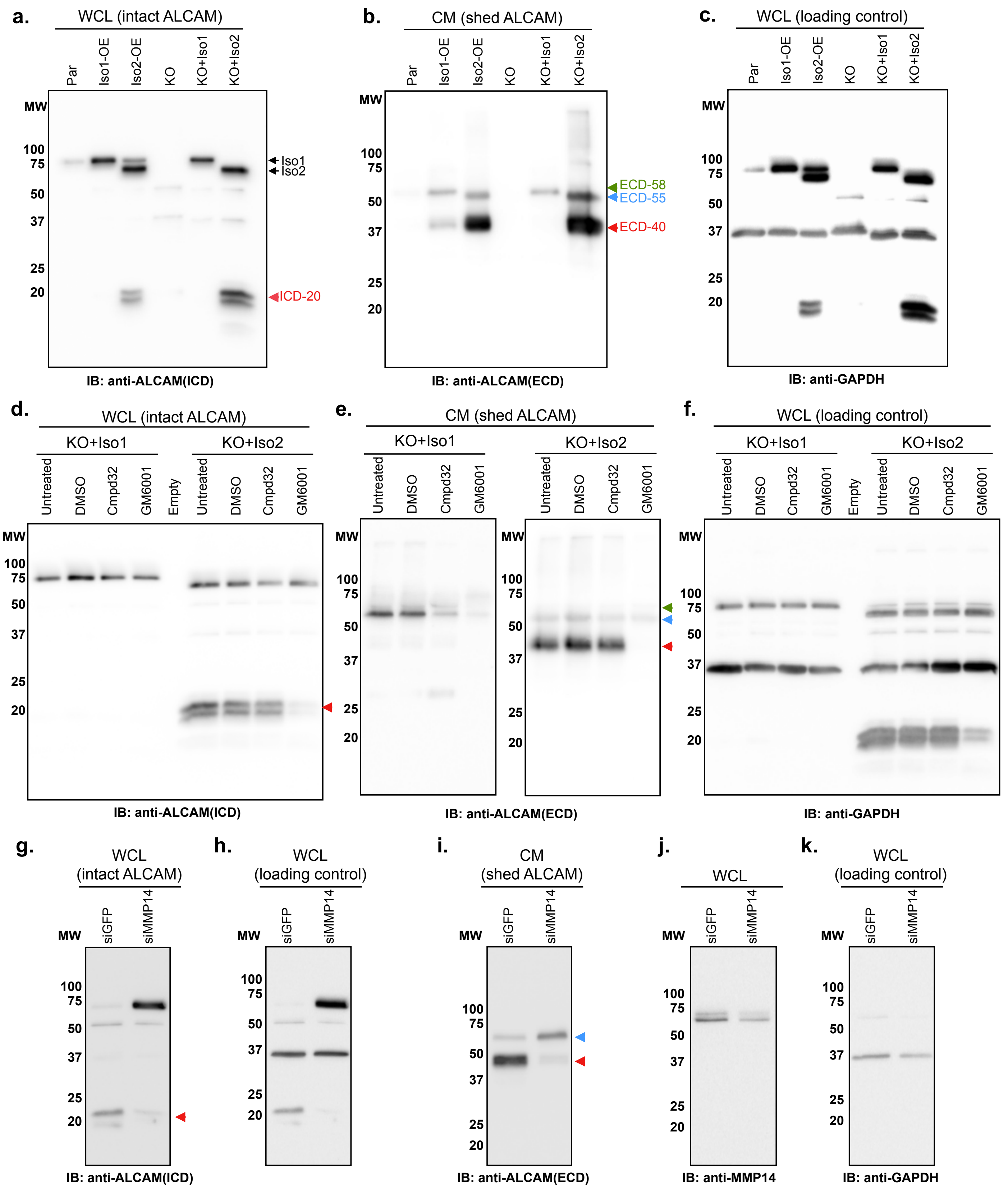
a.
WCL (intact ALCAM)
Par
Iso1-OE
Iso2-OE
KO
KO+Iso1
KO+Iso2
MW
100
75
50
37
25
20
Iso1
Iso2
ICD-20
IB: anti-ALCAM(ICD)
b.
CM (shed ALCAM)
ECD-58
ECD-55
ECD-40
IB: anti-ALCAM(ECD)
c.
WCL (loading control)
IB: anti-GAPDH
d.
WCL (intact ALCAM)
KO+Iso1
KO+Iso2
Untreated
DMSO
Cmpd32
GM6001
Empty
IB: anti-ALCAM(ICD)
e.
CM (shed ALCAM)
IB: anti-ALCAM(ECD)
f.
WCL (loading control)
IB: anti-GAPDH
g.
WCL
(intact ALCAM)
siGFP
siMMP14
IB: anti-ALCAM(ICD)
h.
WCL
(loading control)
i.
CM
(shed ALCAM)
IB: anti-ALCAM(ECD)
j.
WCL
IB: anti-MMP14
k.
WCL
(loading control)
IB: anti-GAPDH

Supplementary Figure S7. Full-length blots corresponding to Figure 3. 5 min exposures are shown. Individual blots are outlined in black. WCL blots were redeveloped with HRP-tagged anti-GAPDH for loading control. ICD: intracellular domain, ECD: extracellular domain. ALCAM fragments generated by shedding are marked as follows: 20 kDa intracellular domain fragment (ICD-20, ◄), 58 kDa extracellular domain fragment (ECD-58, ◄), 55 kDa extracellular domain fragment (ECD-55, ◄), and 40 kDa extracellular domain fragment (ECD-40, ◄). (**a**) Full-length blot corresponding to Figure 3b, left panel. (**b**) Full-length blot corresponding to Figure 3b, right panel. (**c**) Full-length blot corresponding to Figure 3b, loading control. (**d**) Full-length blot corresponding to Figure 3d, left panel. (**e**) Full-length blots corresponding to Figure 3d, right panel. (**f**) Full-length blot corresponding to Figure 3d, loading control. (**g**) Full-length blot corresponding to Figure 3e, left panel. (**h**) Full-length blot corresponding to Figure 3e, left panel loading control. (**i**)Full-length blot corresponding to Figure 3e, middle panel. (**j**) Full-length blot corresponding to Figure 3e, right panel. (**k**) Full-length blot corresponding to Figure 3e, right panel loading control.

Name	Transcript ID	bp	Protein	Biotype	CCDS	UniProt	RefSeq	Flags
ALCAM-001	ENST00000306107.9	4701	583aa	Protein coding	CCDS33810	Q13740	NM_001243281 NM_001627 NP_001230210 NP_001618	TSL:1, GENCODE basic, APPRIS P1
ALCAM-002	ENST00000472644.6	4189	570aa	Protein coding	CCDS58841	Q13740	NM_001243280 NP_001230209	TSL:1, GENCODE basic
ALCAM-007	ENST00000465413.6	2496	344aa	Protein coding	-	H7C543	-	CDS 5' incomplete, TSL:2
ALCAM-005	ENST00000486979.6	1818	532aa	Protein coding	-	F5GXJ9	-	TSL:5, GENCODE basic
ALCAM-004	ENST00000481337.5	1242	No protein	Processed transcript	-	-	-	TSL:2
ALCAM-008	ENST00000460954.1	564	No protein	Processed transcript	-	-	-	TSL:4
ALCAM-006	ENST00000491388.6	2845	No protein	Retained intron	-	-	-	TSL:2
ALCAM-003	ENST00000470756.5	1770	No protein	Retained intron	-	-	NM_001243283	TSL:1
ALCAM-009	ENST00000489178.1	783	No protein	Retained intron	-	-	-	TSL:2

Supplementary Table ST1. ALCAM predicted splice isoform information. CCDS=consensus coding sequence project identification number, TSL=transcript support level, APPRIS P1=APPRIS principal isoform (Ensembl).

Inhibitor	Targets	Concentration
Aprotinin	Serine proteases	0.3 μM
Compound 32	ADAM17	10 μM
E64	Cysteine proteases	10 μM
GM6001	Metalloproteases	10 μM
Leupeptin	Trypsin-like proteases<br>Serine proteases	100 μM
Pepstatin A	Aspartic proteases	1 μM
PMSF	Serine proteases<br>Papain	1 mM

Supplementary Table ST2. Compounds used in protease inhibitor panel (Sup. Fig. S4).

		Par					KO					KO+Iso1					KO+Iso2				
		Untr	Veh	A-Fc	MPi	A-Fc+MPi	Untr	Veh	A-Fc	MPi	A-Fc+MPi	Untr	Veh	A-Fc	MPi	A-Fc+MPi	Untr	Veh	A-Fc	MPi	A-Fc+MPi
Par	Untr		ns	**0.0022**	**<0.0001**	**ns**	ns					**<0.0001**					**<0.0001**				
	Veh			0.0001	<0.0001	0.0119		ns					<0.0001					<0.0001			
	A-Fc				**<0.0001**	**ns**			ns					<0.0001					0.0281		
	MPi					**<0.0001**				<0.0001					<0.0001					<0.0001	
	A-Fc+MPi										<0.0001					0.0003					ns
KO	Untr							ns	**ns**	**0.0141**	**0.0077**	**<0.0001**					**<0.0001**				
	Veh								ns	0.0058	0.0031		<0.0001					<0.0001			
	A-Fc									**0.0001**	**<0.0001**			<0.0001					ns		
	MPi										**ns**				<0.0001					<0.0001	
	A-Fc+MPi															ns					<0.0001
KO+Iso1	Untr												ns	**<0.0001**	**0.0004**	**<0.0001**	**<0.0001**				
	Veh													<0.0001	0.0037	<0.0001		<0.0001			
	A-Fc														**<0.0001**	**ns**			0.0096		
	MPi															**<0.0001**				ns	
	A-Fc+MPi																				0.0002
KO+Iso2	Untr																	ns	**<0.0001**	**<0.0001**	**<0.0001**
	Veh																		<0.0001	<0.0001	<0.0001
	A-Fc																			**<0.0001**	**ns**
	MPi																				**<0.0001**
	A-Fc+MPi																				

Supplementary Table ST3. P-values for Chi-square test for trend of data in Figure 5b. Comparisons discussed in text are bolded.

		Par				Iso1-OE				Iso2-OE			
		Untr	Veh	ALCAM-Fc	GM6001	Untr	Veh	ALCAM-Fc	GM6001	Untr	Veh	ALCAM-Fc	GM6001
Par	Untr		ns	**0.0041**	**0.0002**	**<0.0001**				**<0.0001**			
	Veh			<0.0001	0.0089		<0.0001				0.0362		
	ALCAM-Fc				**<0.0001**			ns				<0.0001	
	GM6001								<0.0001				<0.0001
Iso1-OE	Untr						ns	**<0.0001**	**0.0492**	**0.002**			
	Veh							<0.0001	0.0026		<0.0001		
	ALCAM-Fc								**<0.0001**			**<0.0001**	
	GM6001												**ns**
Iso2-OE	Untr										ns	**<0.0001**	**<0.0001**
	Veh											<0.0001	<0.0001
	ALCAM-Fc												**<0.0001**
	GM6001												

Supplementary Table ST4. P-values for Chi-square test for trend of data in Supplementary Figure S5b. Comparisons discussed in text are bolded.

		Par			KO			KO+Iso1			KO+Iso2		
		Untr	KO CM	Iso2 CM	Untr	KO CM	Iso2 CM	Untr	KO CM	Iso2 CM	Untr	KO CM	Iso2 CM
Par	Untr		0.0298	0.0309	**ns**			**<0.0001**			**<0.0001**		
	KO CM			**ns**		ns			<0.0001			0.0179	
	Iso2 CM						ns			<0.0001			ns
KO	Untr					ns	ns	**<0.0001**			**<0.0001**		
	KO CM						**ns**		<0.0001			0.0002	
	Iso2 CM									<0.0001			0.0013
KO+Iso1	Untr								ns	0.0079	**0.0031**		
	KO CM									**0.0144**		<0.0001	
	Iso2 CM												<0.0001
KO+Iso2	Untr											ns	<0.0001
	KO CM												**0.0015**
	Iso2 CM												

Supplementary Table ST5. P-values for Chi-square test for trend of data in Figure 6b. Comparisons discussed in text are bolded.

		Par				KO				KO+Iso1				KO+Iso2			
		Untr	Veh	ALCAM-Fc	GM6001	Untr	Veh	ALCAM-Fc	GM6001	Untr	Veh	ALCAM-Fc	GM6001	Untr	Veh	ALCAM-Fc	GM6001
Par	Untr		ns	**<0.0001**	**ns**	**<0.0001**				**0.0113**				**0.0094**			
	Veh			<0.0001	ns		<0.0001				ns				0.0339		
	ALCAM-Fc				**<0.0001**			ns				0.0035				0.0005	
	GM6001								0.0058				<0.0001				0.0307
KO	Untr						ns	**ns**	**0.0014**	**<0.0001**				**<0.0001**			
	Veh							ns	0.0004		<0.0001				<0.0001		
	ALCAM-Fc								**0.0002**			ns				0.0342	
	GM6001												<0.0001				<0.0001
KO+Iso1	Untr										ns	**<0.0001**	**ns**	**<0.0001**			
	Veh											<0.0001	<0.0001		0.0209		
	ALCAM-Fc												**<0.0001**			**ns**	
	GM6001																**0.0350**
KO+Iso2	Untr														ns	**<0.0001**	**0.0003**
	Veh															0.0012	<0.0001
	ALCAM-Fc																**<0.0001**
	GM6001																

Supplementary Table ST6. P-values for Chi-square test for trend of data in Figure 7b. Comparisons discussed in text are bolded.
